# Novel outpatient management of mild to moderate COVID-19 spares hospital capacity and safeguards patient outcome: The Geneva PneumoCoV-Ambu study

**DOI:** 10.1371/journal.pone.0247774

**Published:** 2021-03-04

**Authors:** Chloé Chevallier Lugon, Mikaela Smit, Julien Salamun, Meriem Abderrahmane, Olivia Braillard, Mayssam Nehme, Frédérique Jacquerioz Bausch, Idris Guessous, Hervé Spechbach

**Affiliations:** 1 Department of Community Medicine Primary Care and Emergency Medicine, Division of Primary Care Medicine, Geneva University Hospitals, Geneva, Switzerland; 2 Department of Medicine, Division of Infectious Diseases, Geneva University Hospitals, Geneva, Switzerland; 3 Faculty of Medicine, University of Geneva, Geneva, Switzerland; Kaohsuing Medical University Hospital, TAIWAN

## Abstract

**Background:**

Severe Acute Respiratory Coronavirus 2 (SARS-CoV-2), the novel coronavirus that causes coronavirus disease (COVID-19), is creating an unprecedented burden on health care systems across the world due to its high rate of pneumonia-related hospitalizations. This study presents recommendations for the outpatient management of moderate SARS-CoV-2 pneumonia implemented at the Geneva University Hospital, Switzerland, from April 4 to June 30, 2020 and evaluated the impact of these recommendations on patient safety, patient satisfaction, and overall hospital capacity.

**Methods:**

Recommendations for the outpatient management of moderate pneumonia implemented in the Geneva University Hospital (PneumoCoV-Ambu) between April 4 and June 30, 2020, were evaluated prospectively. The primary endpoint was hospitalization. Secondary endpoints were: severity of COVID-19 disease based on a 7-points ordinal scale assessed at 1 and 2 months following SARS-CoV-2 infection; patient satisfaction using a satisfaction survey and the analysis of number of beds and costs potentially averted.

**Results:**

A total of 36 patients with COVID-19-related pneumonia were followed between April 4 and May 5, 2020. Five patients (14%) were hospitalized and none died over a median of 30 days follow-up. The majority of patients (n = 31; 86%) were satisfied with the ambulatory care they received. These novel recommendations for outpatient management resulted in sparing an estimated potential 124 hospital bed-nights and CHF 6’826 per capita averted hospitalization costs over the three months period.

**Conclusions:**

Recommendations developed for the outpatient management of COVID-19-related pneumonia were able to spare hospital capacity without increasing adverse patient outcomes. Widely implementing such recommendations is crucial in preserving hospital capacity during this pandemic.

## Introduction

The novel Severe Acute Respiratory Coronavirus 2 (SARS-CoV-2) and associated coronavirus disease (COVID-19) was declared a pandemic by the World Health Organization (WHO) on March 11, 2020 [1]. It is continuing to propagate worldwide, resulting in unprecedented challenges for health systems across the world through its high hospitalization rate relating to respiratory disease. It is now known that COVID-19 causes clinical syndromes ranging from asymptomatic infections or mild respiratory illnesses to severe pneumonia, acute respiratory distress and death.

Averting hospitalizations is key to preserving health care capacity across countries profoundly affected by COVID-19. Crucial to these efforts is the development and formal evaluation of guidelines on the outpatient management of COVID-19-related pneumonia. Whilst guidelines have been established for the treatment of COVID-19 associated pneumonia in hospitalized patients [2], there is, to date, no consensus on guidelines for outpatient management of COVID-19 pneumonia. The WHO, for example, recommends close monitoring of patients with moderate COVID-19 for signs or symptoms of disease progression and in the presence of risk factors, recommends hospitalization [3]. Yet, the preference across a number of countries has been to admit early the patients who present with moderate or severe illness to a hospital for close observation [2].

In the Canton of Geneva, Switzerland, the health authorities designated the University Hospital of Geneva (HUG), the largest and only public university hospital in the Canton, as the sole dedicated COVID-19 hospital. At the HUG, patients presenting with mild to moderate COVID-19-related pneumonia were not systematically hospitalized but instead were followed as outpatients in order to preserve the health care capacity. Novel recommendations were developed to fit this purpose. This study is an evaluation to determine the impact of these recommendations on patient safety, patient satisfaction and hospital bed-nights averted between April and June 2020.

## Method

### Setting

This study was conducted at the HUG, Switzerland, where in March 2020 the Department of Primary Care Medicine which was put in charge of leading the ambulatory COVID-19 response for the Canton of Geneva. This included setting up dedicated COVID-19 testing centers for the Canton and the development of novel guidelines outlining recommendations for outpatient management [4] and the indication for hospitalization [5].

**Fig 1:**
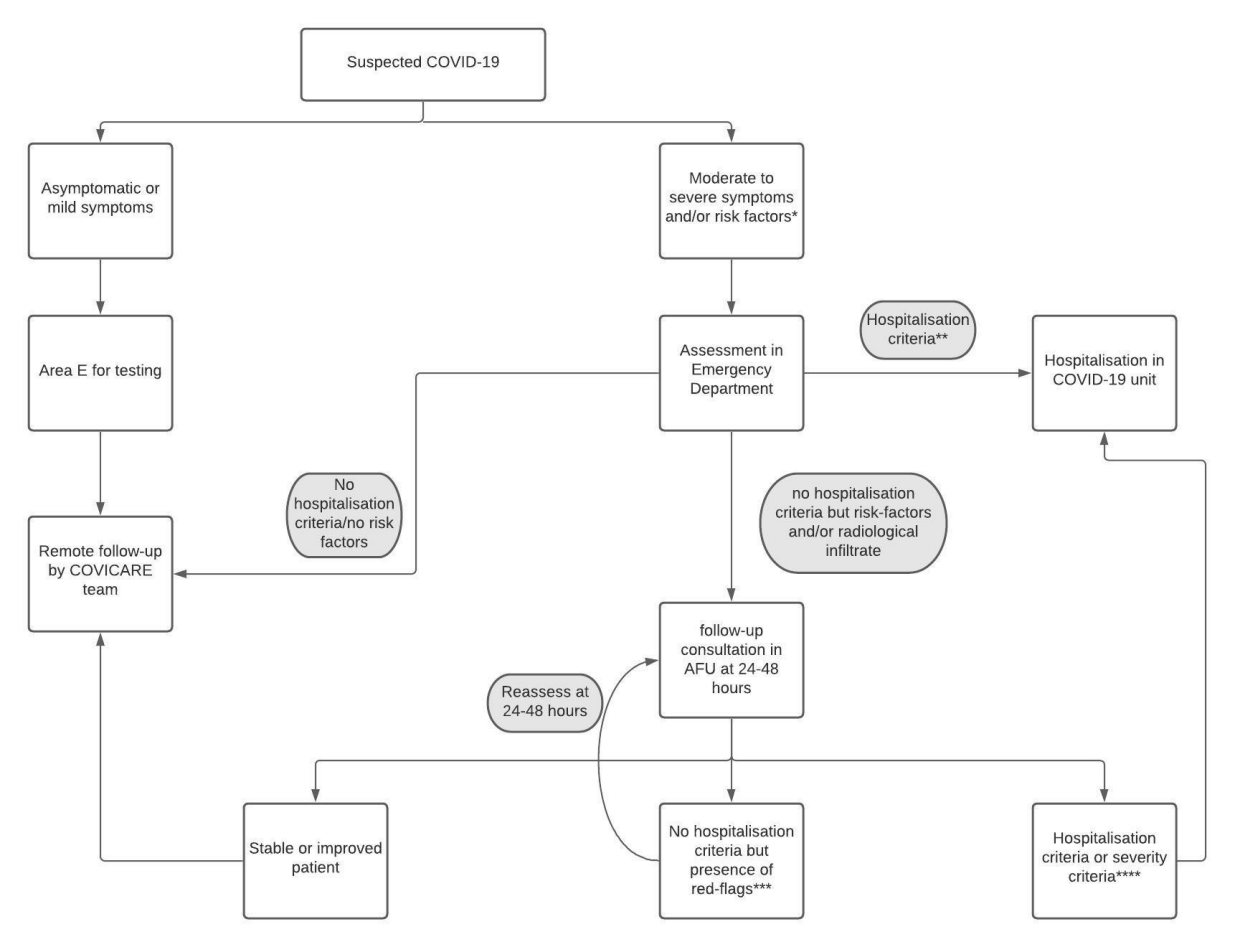
Patient’s trajectory with suspected COVID-19 depending their symptoms. *Risk factors: above 65 years old, hypertension, diabetes, cardiovascular disease, chronic respiratory disease, immunosuppression, cancer. ** Hospitalization criteria: Pneumonia with CURB-65 > = 2, oxygen dependency, sustained tachypnea (RR≥20 min) decompensated comorbidity (ies). *** Red-flags: Cough and/or fever in worsening condition, dyspnea NYHA III, hemoptysis, decreased general condition, ECOG performance status 2–3, Altered state of consciousness, syncope. **** Severity criteria: audible dyspnea, inability to speak (dyspnea NYHA Stage 4), serious decline in general condition (performance status >3).

Two testing centers were set up; “Area E” was designed to test all patients with asymptomatic or mild disease only whilst patients presenting with moderate to severe disease or those with risk factors (specifically—those over 65 years old, or those that had hypertension, diabetes, cardiovascular disease, chronic respiratory disease, immunosuppressive disorder or, cancer) were assessed in the Emergency Department (ED) (Fig 1). All patients presenting for SARS-CoV-2 testing were led to dedicated entrances separate from that of the general population attending the hospital.

Adult patients presenting with mild-to-moderate respiratory symptoms at the dedicated testing centers (Area E and the ED) at the HUG, between April 4^th^ and May 5^th^ 2020, who had a positive RT-PCR test result for SARS-CoV-2 with either a nasopharyngeal or oropharyngeal swab specimens, were included in this analysis. The data collection period was prospective from May 6 to June 30, 2020. The Cantonal Commission for Ethics and Research (CCER) has approved this study and the use of oral consent in April 2020.

### Ethics statement

This trial was approved by the Cantonal Research Ethics Commission (CCER), Geneva, Switzerland (protocol number CCER 2020–01518). The COVICARE team obtained informed verbal consent by telephone for the use of the data at the time of diagnosis. A form was sent to them by message (SMS and email) for them to validate this consent in writing. Verbal consent was documented in a coded database (REDCap). In addition, since March 2020 and the beginning of the outbreak, all HUG screening and emergency webpages contain statements informing healthcare users that their clinical data may be collected and used in clinical studies on COVID-19. Any patient who refuses to allow their data to be used must inform the health care provider at the time of care provision. The data were collected prospectively and recorded in an Excel database by the same team who did the follow up consultation. The data collected were then anonymized in Excel for the statistical analysis. Excel database is stored on a protected network of the hospital. Access to the database is limited by a password (See S1 Dataset).

### Outpatient guidelines

Patients presenting to Area E were tested and then advised to self-isolate at home, where they received a follow-up telephone call from the dedicated COVICARE team [6, 7] (Fig 1). The COVICARE team, composed of primary care physicians and medical students communicated the test results and provided remote ambulatory follow-up to COVID-19 positive patients by phone or video consultations. The COVICARE team was born from a need of the population in the canton of Geneva. Patients were feeling very isolated and alone when they were diagnosed with a mild or moderate disease at home with many general practitioners in Geneva who were not equipped to see COVID positive patients due to a lack of protective equipment and unsuitable ambulatory infrastructure. The COVICARE follow-up consisted of calling patients every 48 hours for the first 10 days following diagnosis, with a standardized interview inquiring about self-reported symptoms or every 24 hours if patients presented a worsening clinical condition (for more details: www.covicare24.com).

Patients presenting to the Emergency Department were first assessed for the presence of the hospitalization criteria (Fig 1). These included: CURB-65 score of more than two points [8], need for oxygen, sustained tachypnea, and any decompensated comorbidities (Fig 1). Patients with such criteria were immediately hospitalized (Fig 1). After testing, those with moderate illness were sent home to await their results. They were invited for an in-person follow-up visit 24 to 48 hours at the COVID Ambulatory Follow-Up Unit (AFU) independent of their test result (Fig 1). Disease severity was categorized using the National Institutes of Health (NIH) and Centre for Disease Control and Prevention (CDC) categorization for SARS-CoV-2 respiratory symptoms [9]. Five categories were used: *asymptomatic or presymptomatic infection* (test positive for SARS-CoV-2 but have no symptoms), *mild illness* (any of various signs and symptoms but without shortness of breath, dyspnea, or abnormal imaging), *moderate illness* (evidence of lower respiratory disease by clinical assessment or imaging and a saturation of oxygen [SaO2] >93% in room air), *severe illness* (respiratory frequency [RF] >30 breaths per minute, [SaO2] ≤93% in room air), *critical illness* (respiratory failure, septic shock, and/or multiple organ dysfunction) [2].

AFU consultations were conducted by a nurse and a senior physician five days a week. Patients were evaluated in a specific room with standardized protective equipment. Surgical mask and smock during the history-taking and gloves, protective glasses and FFP2 mask during the clinical examination. We did not record any cases of contamination of the healthcare team in the professional setting during this period. During the week-end, patients were assessed using the same protocol in the emergency outpatient unit. The AFU consultation included a reassessment of pneumonia severity, based on the evolution of symptoms, significant alterations in their clinical condition, as well as radiological and laboratory findings (Fig 1). The patients’ socio-economic status was also taken into consideration in order to ensure effective isolation at home. Patients presenting with criteria for hospitalization during this follow-up visit were directly sent to a COVID-19 hospital unit from AFU. Those without any hospitalization criteria were sent home with either a second in-patient consultation at AFU at 24 to 48 hours or were handed over to the COVICARE team for remote monitoring over a period of 10 to 15 days (Fig 1).

### Variables collected

Data on demographic variables (age, gender), date of symptom onset and comorbidities were collected in both Area E and the Emergency Department before being coded by the COVICARE team into a protected database. During follow-up consultations at AFU, data on additional symptoms were collected on a dedicated computerized form. These included: dyspnea grading based on the New York Health Association classification [10], anosmia, chest pain, fever, headache and Eastern Cooperative Oncology Group (ECOG) scale performance status [11], a graduation scale used in oncology to classify the functional impairment of a patient from 0 (no impairment) to 5 (death). Risk factors and medical history were graded with the Charlson comorbidity index [12], a score predicting the 10 year mortality of patients with multiple comorbidities. Imaging studies and laboratory results were analyzed to assess the overall health status of each patient, if available.

In addition, the COVICARE team performed a follow-up with all patients 30 days after diagnosis, collecting data on any additional consultations with their General Practitioner, COVID-19 related visits to Emergency Department or hospitalizations within 30 days [7].

All data are available in a S1 Dataset and legends in S1 Appendix.

### Outcomes

The primary endpoint was COVID-19-related hospitalizations or death 30 to 60 days following diagnosis. Secondary outcomes were:

- severity of COVID-19 disease 30 to 60 days after SARS-CoV-2 diagnosis, based on a 7-points ordinal severity scale [3] (1: not hospitalized, no limitation of activities; 2: not hospitalized, limitation of activities; 3: hospitalized, not requiring supplementary oxygen; 4: hospitalized, requiring supplementary oxygen; 5: hospitalized, on non-invasive mechanical ventilation; 6: hospitalized, on invasive mechanical ventilation or ECMO; 7: death);
- Patient satisfaction with the ambulatory management strategies measured by a questionnaire administered by phone two to three months post diagnosis. Satisfaction was evaluated with questions on the patient’s preference for remote monitoring, for hospitalization or other. The questionnaire is available in the S2 Appendix.
- Number of spared hospital bed-nights and averted care costs compared with a strategy where all COVID-19 related pneumonia cases had been hospitalized.

### Cost data

We used data from the HUG Finance Department in order to estimate the average hospitalization cost for patients with pneumonia. The average hospitalization cost per day for pneumonia from the 2018 management accounts varied from CHF 1,057 for the Geriatrics Department to CHF 2,356 for the General Internal Medicine Department (1CHF 〜 1 USD).

For this analysis, we averaged the cost of hospitalization to be CHF 1’706.50 per patient per day. For the duration of hospitalization, we extrapolated from past data for the hospitalization for community-acquired pneumonia as the equivalent data for COVID-19 moderate pneumonia hospitalization at the HUG between April and June 2020 are not yet available. The mean hospitalization stay for community acquired pneumonia varied between three to five days, depending on the age and patient comorbidities. The average length of stay is four days, which resulted in an average hospitalization cost of CHF 6 826 per patient.

Outpatient costs were calculated using the cohort of patients consulting the dedicated testing center based on the Emergency Department’s billing service. The average costs were between CHF 500–1000 and included nursing triage, medical consultation and additional tests (laboratory tests, x-rays). In addition to outpatient care, we calculated remote follow-up by the COVICARE team; every 48 hours over a 10 day period (CHF 50 per call) and one in-person follow-up consultation in the dedicated COVID-19 ambulatory care unit (CHF 150). This resulted in an average outpatient cost of CHF 1 150 per patient.

### Statistical analysis

In this observational study, we prospectively evaluated a sample of 36 patients who presented to Area E or the Emergency Department and met the criteria as outlined previously. We used descriptive statistics, frequency distributions, and chi-square analyses to characterize the sample and we performed a cursory cost estimate reflecting savings based on in-patient versus outpatient care recommendations.

## Results

### Participants

**Fig 2:**
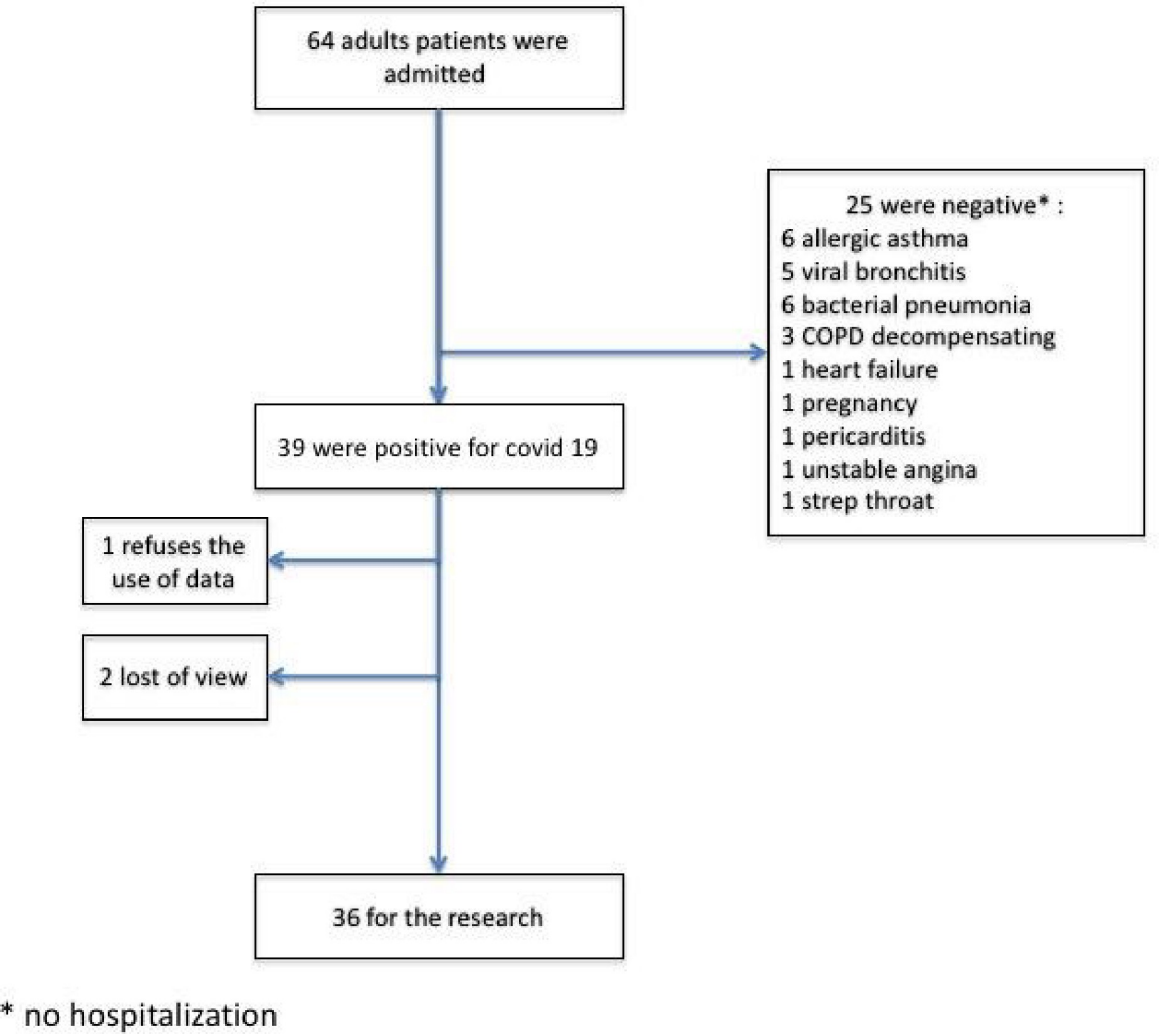
Flow chart of study design.

During the study period, 64 patients were identified as having suspected SARS-CoV-2 moderate pneumonia in the dedicated testing center and benefited from a follow up consultation with either the AFU and/or COVICARE. Of these, 25 patients tested negative for COVID-19 and had an alternative diagnosis (6 with asthma, 6 with bacterial pneumonias, 5 with non- COVID viral bronchitis, 3 decompensations of COPD, 1 with heart failure, 1 with pregnancy, 1 with pericarditis, 1 with unstable angina and 1 with streptococcal pharyngitis). Of note, the 6 patients with bacterial pneumonia, defined as radiological unilateral condensation, were not hospitalized. The remaining 39 patients tested positive for SARS-CoV-2 and had pneumonia. Of the 39 patients, one did not consent to take part in the study and two were lost to follow-up, resulting in 36 patients being included in the analyses (Fig 2).

Demographic and clinical characteristics of the 36 patients are presented in Table 1. The median age was 46 years old (range 21–66), and 21 (56%) were male. The majority (69%) were aged between 40 and 65 years old and only one (3%) patient was above 65 years old.

**Table 1:** Characteristics of patients with COVID-19-related pneumonia.

	All patients N = 36 (100%)	Non hospitalized N = 31 (86%)	Hospitalized N = 5 (14%)
**Gender**			
Male	20 (56)	17 (55)	3 (60)
Female	16 (44)	14 (45)	2 (40)
**Age**			
Mean (years SD)	46 (10,56)	44,4 (10,2)	56 (6,6)
Under 40 years old	10 (28)	10 (32)	0 (0)
Between 40 and 65 years old	25 (69)	20 (65)	5 (100)
Above 65 years old	1 (3)	1 (3)	0 (0)
**Co-morbidities and risk factors**			
Asthma	7 (19)	6 (19)	1 (20)
Active smoker	4 (11)	4 (13)	0 (0)
Cancer	1 (3)	1 (3)	0 (0)
Diabetes	2 (6)	2 (6)	0 (0)
Former smoker	4 (11)	4 (13)	0 (0)
Hypertension	2 (6)	0	2 (40)
Immunocompromised	1 (2,8)	1 (3)	0 (0)
Obesity	1 (2.8)	1 (3)	0 (0)
Obstructive sleep apnea	2 (5,6)	2 (6)	0 (0)
**Charlson Comorbidity Index**			
• Score 0	27 (72,2)	24(77)	2(40)
• Score 1	9 (25)	6 (19)	3 (60)
• Score 2	1 (2,8)	1 (3)	0 (0)
• Score 3 and above	0 (0)	0 (0)	0 (0)
**Pneumonia symptoms**			
Dyspnea I	10 (27,8)	10 (32)	0 (0)
Dyspnea II	10 (27,8)	9(29)	1 (20)
Dyspnea III	15 (41,6)	12(39)	3 (60)
Dyspnea IV	1 (2,8)	0 (0)	1 (20)
**Other symptoms**			
Anosmia	8 (22,2)	7 (22,6)	1 (20)
Fever	5 (13)	3 (9,7)	2 (40)
Headache	12 (33,3)	12 (38,7)	0 (0)
Retro sternal pain	13 (36,1)	11 (35,5)	2 (40)
**CURB-65 score**			
• Score 0	32 (88,8)	29(93,5)	3(60)
• Score 1	4 (11.2)	2 (6,5)	2 (40)
• Score 2 or above	0 (0)	0 (0)	0 (0)
**ECOG Performance status**			
• 0	7 (19,4)	6 (19,3)	1 (20)
• 1	13 (36,1)	13 (41,9)	0 (0)
• 2	10 (27,8)	9 (29)	1 (20)
• 3	5 (13)	3 (9,7)	2 (40)
• 4	1 (2.8)	0	1 (20)
**Standard thoracic radiograph performed**	34 (94,4)	29 (94)	5 (100)
**Interstitial infiltrate found on radiograph**	23 (63,9)	18 (58)	5 (100)

Regarding comorbidities, 24.6% suffered from pulmonary disease (asthma and obstructive sleep apnea), 6% diabetes, 6% hypertension, 3% cancer, 2.8% were immunocompromised, and 2.8% were obese. On the Charlson Comorbidity Index, which predicts 10-year mortality, 27 patients (72%) had zero points, nine (25%) had one point and one (2.8%) had two points. Amongst the five patients who were subsequently hospitalized, two had zero points and three had one point.

During follow-up, all patients reported shortness of breath and 25 (70%) presented with NYHA stage II (slight limitation) or III dyspnea (shortness of breath during limited activity). Eight (22.2%) declared anosmia at follow up, twelve (33.3%) complained of headaches and 13 (36,1%) presented with chest pain. Only five (13%) had a fever at follow up. The CURB-65 score was calculated for each patient during consultation. The majority had zero point (88,8%) and four (11,2) had one point. In those four, two were hospitalized. One patient had blood urea above 7 mmol per liter and one had diastolic blood pressure below 60mmHg. None had a CURB-65 above one point. We assessed the ECOG scale performance status of each patient during the follow-up consultations. Seven (19,4%) had no impairment (scale 0), 13 (36,1%) had some restriction in physical activity (scale 1), ten (27,8%) were capable of all self-care but unable to carry out any work activities (scale 2), five (13%) were capable of only limited self-care and were confined to bed or chair for more than 50% of waking hours (scale 3) and one (2,8%) was completely disabled (scale 4). None were deceased (scale 5). Nearly all patients had had a chest X-ray (94,4%) at initial evaluation, which had revealed bilateral interstitial infiltrates in 23 patients (64%).

**Fig 3:**
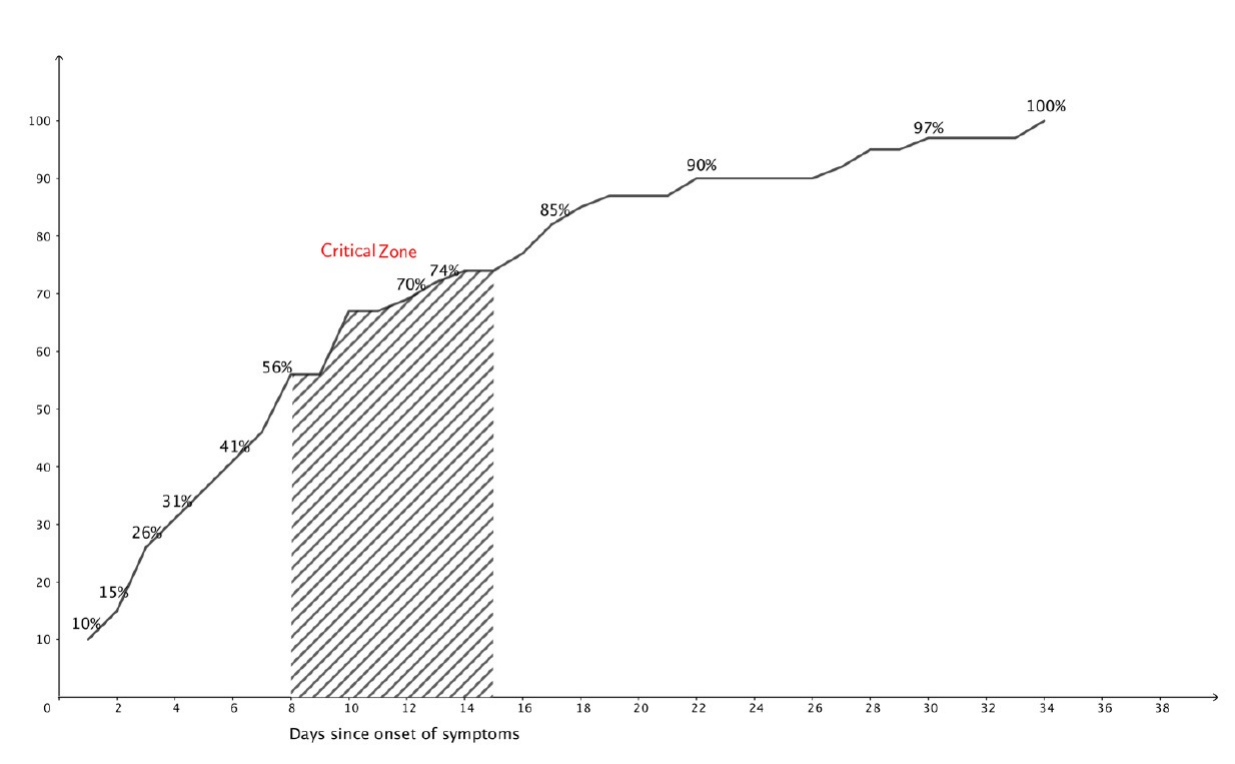
Cumulative frequency: Number of days between onset of symptoms and follow-up consultation at ambulatory follow-up unit (AFU). a) The critical zone corresponds with the highest peak of complications.

Fig 3 demonstrates the follow-up time, specifically the number of days between onset of symptoms and the first consultation at AFU. The majority of patients were seen within the first week after the onset of symptoms and 25% within the first 3 days. At 14 days, 75% had had a consultation with a physician, with 14 days being the critical zone for respiratory decompensation and need for intensive care unit (ICU) transfer [13, 14].

### Outcomes

In this outpatient cohort, only five patients (14%) required secondary hospitalization (Table 2). These hospitalizations occurred within 30 days of diagnosis and were the result of worsening of symptoms or new need for oxygen therapy. Amongst these five patients, two patients were hospitalized within 48 hours of diagnosis directly from the initial AFU assessment, two were transferred from AFU to the emergency room for further investigations and were subsequently hospitalized and one was hospitalized between two scheduled consultations at AFU.

**Table 2:** Overview of hospitalized patients.

Patient ID	Time to hospital^a^ (days)	Hospitalization criteria	Need for oxygen	Inpatient destination	hospital bed nights (days)	Outcome
1	13	Dyspnea grade IV	yes	Intermediate care unit for non-invasive ventilation	15	discharged
		Saturation <93%				
2	11	Dyspnea grade IV	yes	COVID unit (internal medicine)	14	discharged
		Saturation <93%				
3	10	Asthenia	no	COVID unit (internal medicine)	2	discharged
		Dyspnea grade III				
4	9	Dyspnea grade IV	yes	COVID unit (internal medicine)	5	discharged
		Saturation <93%				
5	3	Pleural effusion	no	COVID unit (internal medicine)	2	discharged

^a^ days from onset of first symptoms of COVID-19 to hospitalization

Amongst the hospitalized patients, two were hospitalized out of precaution and only needed close monitoring, two patients needed oxygen therapy and one needed intermediate care with non-invasive ventilation (Table 2). The majority of hospitalization (4/5) occurred within three days of their initial consultation at the dedicated testing center—two within three days, two within two days and one after seven days—but all within ten days of onset of symptoms (Table 2). There were no recorded deaths during the follow-up period.

Most patients presented with grade 1 or 2 disease severity on the SARS-CoV-2 pneumonia scale, 30 to 60 days after diagnosis. The hospitalized patients presented a higher grade of disease severity, respectively two patients with grade 3 (no need for oxygen), two patients with grade 4 (oxygen therapy needed) and one with grade 5 (non-invasive mechanical ventilation).

### Patient satisfaction and costs averted

The majority (30 patients; 84%) were pleased to have remained as outpatient a result of these novel recommendations and dedicated follow-up. Six patients (16%) would have preferred to be admitted to the hospital; two because of the anxiety of spreading the disease to their entourage, and four patients who were subsequently hospitalized would have preferred to be hospitalized immediately.

These novel recommendations for the outpatient management of COVID-19-related mild and moderate pneumonia averted a total of 31 hospitalizations over the period of two months. At an average hospitalization cost of CHF 6 826 per patient, guidelines recommending hospitalization of all mild to moderate cases would have resulted in CHF 245 736 total care costs for the 36 patients. Instead, these novel recommendations resulted in CHF 71 030 total care costs (CHF 36 900 outpatient costs and CHF 34 130 in-patient costs) and thus CHF 174 706 savings. At an average of four nights per hospitalization, the new recommendations also saved 124 hospital bed-nights.

## Discussion

In this study we present novel recommendations for the outpatient management of mild to moderate COVID-19-related pneumonia in one of the largest public hospitals in Europe.

Our study showed that no deaths and no excess hospitalization occurred as a result of implementing these novel recommendations. With our strategy, costly and unnecessary hospitalizations were avoided and hospital capacity was therefore spared. This approach was well accepted by patients, who reported a high level of satisfaction with the care they received.

In USA, numerous studies show that home-health care is better cost-wise, and for the patients and health professionals for the management of chronic disease patients who frequently use the Emergency Department [13, 14]. Our outpatient management strategy are in the same spirit, to preserve patients at home without jeopardizing their health and saving unnecessary hospitalizations.

Our recommendations allows patients to be seen every two days in AFU and three quarters of patients were seen within 14 days after the onset of symptoms. Other studies show that the critical period for respiratory decompensation is between the eighth and fifteenth day [15–17]. This emphasizes the fact that our recommendations have a patient-oriented follow-up. These recommendations were able to triage patients in a timely manner and anticipate the need for hospitalization before the onset of respiratory distress and a clinically critical state. Other studies confirm that hospitalizing patients before the onset of critical illness and respiratory decompensation improves patient’s outcome and lower mortality [17, 18].

The first wave of this pandemic highlighted the need for the development of novel and tailored guidelines for the management of both hospitalized and ambulatory patients suffering from COVID-19. Guidelines are crucial to ensure high standards of care for patients, positive patient outcomes and the safeguarding of healthcare capacity. The latter has been highlighted as a particular challenge in the management of the COVID-19 pandemic. The first wave in spring of 2020, highlighted how even the strongest healthcare systems with some of the highest ICU capacity per capita, such as Northern Italy or the USA [19, 20] struggled to cope under the sheer influx of patients. It is highly likely that the second wave, on whose cusp we now stand, will result in an even greater challenge for healthcare systems across the world, given that countries are unlikely to enforce the same stringent lockdown measures as during the first wave, perhaps in order to uphold national economies.

Key to tackling a second wave will be the sharing of lessons learned during the first wave across different regions and settings. Only in doing so, can we shorten the learning curve in our fight against this novel virus. Our hope is that in sharing the approach used in the Canton of Geneva this may result in the adoption of these or similar outpatient guidelines for the management of mild to moderate pneumonia and thus help safeguard hospital capacity in those settings. This study reinforces our management strategies and we hope to extend these guidelines further.

To our knowledge, recommendations for the outpatient management of mild to moderate COVID-19-related pneumonia have not been reported so far. By using a strict and uniform protocol, the study was able to ensure reproducibility in the standard of care across all patients, while the long and detailed follow-up ensured robust collection of all relevant clinical data. The strengths of this management is that we offered a viable home-health care options to hospitalization. The patients were remotely follow-up by telephone or with in-patient visit at AFU and were reminded at each consultation of the barrier gestures (hand washing, mask using). In addition, each visit was an opportunity to remind them of the alarm symptoms they should be aware of, thus avoiding delays in treatment.

This study presents several limitations. Firstly, the sample size is small, limiting the statistical power of the study. Evaluation of the recommendations across a number of regions and centers in the second wave would lead to further data on individual- and population-level outcomes. The cohort of patients were young, but they too had comorbidities. It is difficult to correlate this population with elderly citizen with various comorbidities but we could extend these findings with older people without prior medical condition and have a better immunity to viral respiratory illnesses. Secondly, the guidelines were implemented and evaluated at a time of reduced hospital capacity, where the healthcare system was rolled-back to its essential functions only in order to provide maximum capacity for COVID-19 care. Evaluation of these recommendations during a second wave, when hospital capacity is unlikely to be rolled-back to the same extend as during the first wave, will provide further insight into operational and practical implications of the guidelines. For example, it is likely that avoiding unnecessary hospitalizations will be even more crucial and that even fewer healthcare personnel will be available to staff the relevant units. Finally, evaluation of the effect of the recommendations on healthcare capacity and costs was limited by the sparse data available on COVID-19 specific data on hospitalization costs and number of hospitalization days. Additional data should become available after the second wave, which would allow an update of these results.

## Conclusion

Recommendations developed for the outpatient management of COVID-19 moderate pneumonia were able to spare hospital capacity without increasing adverse patient outcomes. Implementing such recommendations more widely, will likely be crucial to preserving hospital capacity during a second wave.

## Supporting information

S1 Dataset (PDF)

S1 Appendix Codes and legend. (PDF)

S2 Appendix Satisfaction survey for outpatient COVID-19. (PDF)
