# Supplementary material for: Novel outpatient management of mild to moderate COVID-19 spares hospital capacity and safeguards patient outcome: The Geneva PneumoCoV-Ambu study
Source: PLoS One. 2021 Mar 4;16(3):e0247774. doi: 10.1371/journal.pone.0247774 (PMC7932514; doi:10.1371/journal.pone.0247774)
Supplement: S1 Dataset — (PDF) [file pone.0247774.s001.pdf]

Patient	Age	Gender	1st consult	1st symptoms	2nd consult	RT PCR SARS-CoV-2	Type of comorbidities	Charlson scale	temperature >38.5°C	Headaches	Chest pain	Loss of smell	ECOG	NYHA	CURB-65	Antibiotics
1	2	1	03.2020	03.2020	04.2020	1	asthma	0	0	0	0	0	2	3	0	1
4	5	1	04.2020	03.2020	04.2020	1	none	0	1	0	0	1	3	4	0	1
7	2	0	04.2020	03.2020	04.2020	1	none	0	1	0	1	0	1	3	0	1
9	4	1	04.2020	03.2020	04.2020	1	depression	0	0	1	1	0	3	3	0	1
10	3	1	04.2020	03.2020	04.2020	1	former smoker	0	1	1	1	1	2	3	0	1
12	3	1	04.2020	03.2020	04.2020	1	none	0	0	1	0	0	1	1	0	1
14	1	0	04.2020	03.2020	04.2020	1	asthma	1	0	1	0	1	1	1	0	0
17	3	0	04.2020	04.2020	04.2020	1	asthma	1	0	1	1	0	1	1	0	0
18	4	0	03.2020	03.2020	04.2020	1	asthma	1	0	0	0	0	4	3	1	0
19	2	0	04.2020	03.2020	04.2020	1	none	0	0	1	0	0	1	2	0	1
20	5	0	04.2020	03.2020	04.2020	1	OSAS	1	0	0	1	0	3	3	0	1
21	3	1	04.2020	03.2020	04.2020	1	none	0	0	0	0	0	2	3	0	1
26	3	1	04.2020	03.2020	04.2020	1	asthma	1	0	0	0	0	1	2	0	0
28	4	1	04.2020	04.2020	04.2020	1	hypertension	0	1	0	1	0	2	3	1	0
29	5	1	04.2020	04.2020	04.2020	1	former smoker	1	0	0	0	0	2	3	1	0
32	4	1	04.2020	03.2020	04.2020	1	obesity, former smoker	0	0	0	0	0	1	2	0	1
34	4	1	04.2020	04.2020	04.2020	1	former smoker	0	0	0	1	0	2	3	0	0
36	4	1	04.2020	03.2020	04.2020	1	none	0	0	0	0	0	0	1	0	0
37	2	1	04.2020	03.2020	04.2020	1	none	0	0	0	0	0	1	2	0	0
39	3	0	04.2020	03.2020	04.2020	1	none	0	0	1	0	0	2	3	0	1
40	3	1	04.2020	03.2020	04.2020	1	none	0	0	0	0	0	0	1	0	1
41	2	1	04.2020	03.2020	04.2020	1	none	0	0	0	0	0	1	2	0	0
42	3	0	04.2020	04.2020	04.2020	1	immunosuppresion	1	0	0	0	0	2	3	0	0
44	3	1	04.2020	04.2020	04.2020	1	hypertension	1	0	0	0	0	0	2	0	1
45	5	0	04.2020	04.2020	04.2020	1	cancer	2	0	0	0	0	0	1	0	0
46	4	1	04.2020	04.2020	04.2020	1	hypercholesterolemia	0	0	0	0	0	1	1	0	1
49	4	1	04.2020	03.2020	04.2020	1	none	0	0	0	1	1	2	3	0	0
50	2	1	04.2020	04.2020	04.2020	1	none	0	0	0	1	1	0	1	0	1
51	4	0	04.2020	04.2020	04.2020	1	active smoker	0	0	0	0	1	1	2	0	1
52	4	0	04.2020	04.2020	04.2020	1	asthma, obesity	0	1	1	0	0	3	3	0	0
56	2	0	04.2020	04.2020	04.2020	1	none	0	0	0	0	1	1	2	0	0
58	3	0	04.2020	03.2020	04.2020	1	OSAS, diabetes, obesity	0	0	1	0	0	1	2	0	0
60	3	0	04.2020	04.2020	04.2020	1	active smoker	0	0	0	0	0	0	1	0	0
61	2	0	03.2020	03.2020	04.2020	1	active smoker	0	0	1	0	1	2	3	1	1
62	2	1	04.2020	03.2020	04.2020	1	asthma, active smoker	0	0	1	0	0	0	1	0	0
64	3	0	03.2020	03.2020	04.2020	1	diabetes	1	0	1	0	0	3	2	0	0

Patient	Type of antibiotics	Days of antibiotics	Xray done	Interstitial infiltrate	Laboratory done	C-RP value (mg/l)	Follow up	Decision at follow up	Severity of COVID-19	Hospitalization's date	Satisfaction	Consent
1	1	1	1	1	1	9	0	NA	2		1	1
4	2	2	1	1	1	60	2	4	5	5.04 - 20.04	0	1
7	1	2	1	1	1	12	0	NA	1		1	1
9	1	3	1	1	1	30	0	NA	2		0	1
10	1	3	1	1	1	83	2	NA	2		1	1
12	3	5	1	1	1	21	2	NA	1		1	1
14	NA	NA	1	0	0	NA	0	NA	1		1	1
17	NA	NA	1	0	1	7	0	NA	1		1	1
18	NA	NA	1	1	1	50	4	4	4	6.04 - 20.04	1	1
19	1	5	1	1	1	14	0	NA	1		1	1
20	2	5	1	1	1	8	4	4	3	6.04 - 08.04	1	1
21	1	7	1	1	1	187	2	2	2		1	1
26	NA	NA	1	0	1	13	0	NA	1		1	1
28	NA	NA	1	1	1	31	1	4	4	10.04 - 15.04	0	1
29	NA	NA	1	1	1	90	2	2	2		1	1
32	3	3	1	1	1	19	0	NA	1		1	1
34	NA	NA	1	1	1	37	2	2	2		1	1
36	NA	NA	1	1	1	104	0	NA	1		1	1
37	NA	NA	1	0	1	5	0	NA	1		1	1
39	4	3	1	1	1	NA	1	1	2		0	1
40	1	6	1	1	1	5	0	NA	1		1	1
41	NA	NA	1	1	1	5	1	1	1		1	1
42	NA	NA	1	0	1	5	0	NA	2		1	1
44	2	5	1	1	1	74	4	4	3	16.04 - 18.04	0	1
45	NA	NA	1	1	1	6	2	1	1		1	1
46	1	5	1	1	1	18	1	1	1		1	1
49	NA	NA	1	0	1	5	1	1	2		1	1
50	2	5	1	0	1	130	1	2	1		1	1
51	2	5	1	1	1	65	1	1	1		1	1
52	NA	NA	1	1	1	5	1	1	2		1	1
56	NA	NA	1	0	1	5	0	NA	1		1	1
58	NA	NA	1	0	1	3	0	NA	1		1	1
60	NA	NA	0	0	0	NA	0	NA	1		1	1
61	4	3	1	0	1	5	0	NA	2		0	1
62	NA	NA	1	0	1	0	0	NA	1		1	1
64	NA	NA	0	0	0	5	0	NA	2		1	1
