# Supplementary material for: Novel outpatient management of mild to moderate COVID-19 spares hospital capacity and safeguards patient outcome: The Geneva PneumoCoV-Ambu study
Source: PLoS One. 2021 Mar 4;16(3):e0247774. doi: 10.1371/journal.pone.0247774 (PMC7932514; doi:10.1371/journal.pone.0247774)
Supplement: S1 Appendix — (PDF) [file pone.0247774.s002.pdf]

Codes and Legend

Age	1= Range 20-30; 2= Range 30-40; 3= Range 40-50
	4= Range 50-60; 5= Range 60-70
Gender	Male = 1
Yes	1
Positive	1
NA	Non applicable/non available
ECOG Performance status	0 = no limitation
	1= limited but light work possible
	2= limited for light work
	3= bed rest 50% of the time
	4= hospitalization
	5= dead
NYHA dypnea	1= no limitation
	2= limitation with intense effort
	3= limitation with small effort
	4= dypsnea at rest
CURB-65 score	1pt confusion
	1pt urea >7mmol/l
	1pt Respiration rate >30/min
	1pt Blood pressure systolic >90mmHg or diastolic < 60mmHg
	1pt >65 years old
Type of antibiotics	1=amoxicilline
	2= amoxicilline/clavulanic acid
	3= doxycycline
	4=other
Follow up	0= primary care physician/Covicare
	1= AFU at 48h
	2= AFU at 24h
	3= transfert to ED
	4= direct hospitalization
Decision at follow up	1= return home
	2= enhance follow-up
	3= transfert to ED
	4= hospitalization
Severity of COVID-19 disease	1= not hospitalised no limitation of activities
	2= not hospitalised, limitation of activities
	3= hospitalised, not requiring supplementary oxygen
	4= hospitalised, requiring supplementary oxygen
	5= hospitalised, on non-invasive mechanical ventilation
	6= hospitalised, on invasive mechanical ventilation or ECMO
	7= death
