# Supplementary material for: Novel outpatient management of mild to moderate COVID-19 spares hospital capacity and safeguards patient outcome: The Geneva PneumoCoV-Ambu study
Source: PLoS One. 2021 Mar 4;16(3):e0247774. doi: 10.1371/journal.pone.0247774 (PMC7932514; doi:10.1371/journal.pone.0247774)
Supplement: S2 Appendix — (PDF) [file pone.0247774.s003.pdf]

Hôpitaux Universitaires Genève

CoviCare

Satisfaction survey for outpatient COVID-19

Question:

During your medical treatment for COVID-19, were you satisfied with the ambulatory care provided by the specialized follow-up unit?

- Yes, I am satisfied
- No, I would have preferred to be hospitalize
- Other

Questionnaire de satisfaction pour les patients COVID-19

Question:

Lors de votre diagnostique de la maladie COVID-19, avez-vous été satisfait de la prise en charge ambulatoire avec l'unité de suivi spécialisée ?

- Oui, je suis satisfait
- Non, j’aurais préféré être hospitalisé(e)
- Autre
